# Detecting the Multiomics Signatures of Factor-Specific Inflammatory Effects on Airway Smooth Muscles

**DOI:** 10.3389/fgene.2020.599970

**Published:** 2021-01-13

**Authors:** Yu-Hang Zhang, Zhandong Li, Tao Zeng, Lei Chen, Hao Li, Tao Huang, Yu-Dong Cai

**Affiliations:** ^1^School of Life Sciences, Shanghai University, Shanghai, China; ^2^Channing Division of Network Medicine, Brigham and Women’s Hospital, Harvard Medical School, Boston, MA, United States; ^3^College of Food Engineering, Jilin Engineering Normal University, Changchun, China; ^4^Bio-Med Big Data Center, CAS Key Laboratory of Computational Biology, CAS-MPG Partner Institute for Computational Biology, Shanghai Institute of Nutrition and Health, Chinese Academy of Sciences, Shanghai, China; ^5^College of Information Engineering, Shanghai Maritime University, Shanghai, China; ^6^Key Laboratory of Tissue Microenvironment and Tumor, Shanghai Institute of Nutrition and Health, Chinese Academy of Sciences, Shanghai, China

**Keywords:** smooth muscles, multiomics signatures, Monte Carlo feature selection, machine learning, rule learning

## Abstract

Smooth muscles are a specific muscle subtype that is widely identified in the tissues of internal passageways. This muscle subtype has the capacity for controlled or regulated contraction and relaxation. Airway smooth muscles are a unique type of smooth muscles that constitute the effective, adjustable, and reactive wall that covers most areas of the entire airway from the trachea to lung tissues. Infection with SARS-CoV-2, which caused the world-wide COVID-19 pandemic, involves airway smooth muscles and their surrounding inflammatory environment. Therefore, airway smooth muscles and related inflammatory factors may play an irreplaceable role in the initiation and progression of several severe diseases. Many previous studies have attempted to reveal the potential relationships between interleukins and airway smooth muscle cells only on the omics level, and the continued existence of numerous false-positive optimal genes/transcripts cannot reflect the actual effective biological mechanisms underlying interleukin-based activation effects on airway smooth muscles. Here, on the basis of newly presented machine learning-based computational approaches, we identified specific regulatory factors and a series of rules that contribute to the activation and stimulation of airway smooth muscles by IL-13, IL-17, or the combination of both interleukins on the epigenetic and/or transcriptional levels. The detected discriminative factors (genes) and rules can contribute to the identification of potential regulatory mechanisms linking airway smooth muscle tissues and inflammatory factors and help reveal specific pathological factors for diseases associated with airway smooth muscle inflammation on multiomics levels.

## Introduction

Smooth muscles are a specific muscle subtype that is widely identified in the tissues of internal passageways, such as vessels, and internal organs, including the lungs and intestines. This type of muscle has the capacity for controlled or regulated contraction and relaxation. Various types of smooth muscles are distributed all over the human body. Airway smooth muscle is a unique smooth muscle type that constitutes the effective, adjustable, and reactive wall covering most of the entire airway from the trachea to lung tissues (Chung, 2000; Lam et al., 2019). Similar to that of other smooth muscles, the coupling of excitation and contraction is the basic approach of airway smooth muscles to realize their unique basic biological function: maintaining the normal and effective ventilation of the lungs (Cieri, 2019).

Airway smooth muscle is regulated by various internal and external factors to maintain the balance required for pulmonary oxygen exchange (Dahl et al., 2018; Reyes-García et al., 2018). Cytokines, such as IL-13 and IL-17, have been confirmed to participate in the regulation of airway smooth muscles (Pascoe et al., 2017; Ba et al., 2018; Zhang et al., 2019; Koziol-White et al., 2020). A systematic analysis of human airway smooth muscle cells (ASMCs) has confirmed that interleukins, including IL-13 and IL-4, participate in the regulation of the hypo-responsiveness of smooth muscle subtypes (Koziol-White et al., 2020). IL-17 has been confirmed to participate in the typical inflammatory reactions of ASMCs (Bexiga et al., 2018; Thompson et al., 2018). The identification of IL-17 together with multiple interleukins as candidate regulators validates the specific contributions of interleukins to the actions of ASMCs.

As discussed above, interleukins, such as IL-13 and IL-17, are functionally correlated with the biological processes of ASMCs, and interactions between interleukins and ASMCs may also be correlated with various diseases. Asthma is a typical respiratory inflammatory disease that has been widely reported to be functionally correlated with airway smooth muscles in an inflammatory environment (Bousquet et al., 2000; Salter et al., 2017; Ramakrishnan et al., 2019; Tliba and Panettieri, 2019). For example, the migration of human airway smooth muscles has been confirmed to be regulated by cytokines, including IL-13 and IL-17, and further contribute to the pathogenesis of asthma (Salter et al., 2017). Moreover, infection with SARS-CoV-2, which caused the worldwide COVID-19 pandemic, involves airway smooth muscles and their surrounding inflammatory environment (Frohman et al., 2020; Sungnak et al., 2020). Therefore, airway smooth muscles and related inflammatory factors (like interleukins) may play an irreplaceable role in the initiation and progression of several severe diseases. Studies on the interactions between airway smooth muscles and related interleukins and the detailed contributions of interleukins to the biological or pathological activation of ASMCs may contribute to the explanation of the detailed pathogenesis of inflammatory pulmonary diseases and help the identification of potential effective biomarkers for drug discovery and treatment improvement.

Many previous studies have attempted to reveal the potential relationships between interleukins and ASMCs at different omics levels. Recently, a specific study on the relationships between asthma-promoting cytokines (IL-13 and IL-17) and ASMCs tried to identify key regulatory factors on the transcriptomics and epigenetics levels. Researchers identified 225 genes around differentially methylated regions by using independent IL-13 and IL-17 and combined interleukins and 2014 differentially expressed transcripts by comparing different cytokine-stimulated groups (Thompson et al., 2019). However, the continued existence of numerous false-positive optimal genes/transcripts cannot reflect the actual effective biological mechanisms underlying interleukin-based activation effects on airway smooth muscles. In this study, on the basis of newly presented computational approaches based on machine learning, we first identified specific regulatory factors (genes) that contribute to the activation and stimulation of airway smooth muscles by IL-13, IL-17, or the combination of both interleukins on the epigenetic and/or transcriptional levels. Next, we also established a series of rules based on essential genes that contribute to distinguishing quiescent and interleukin (either independent or combined)-activated ASMCs in a quantitative manner. Our results, including detected discriminative genes and quantitative rules, corresponding to different patterns, can contribute to the identification of potential regulatory mechanisms underlying interactions between airway smooth muscle tissues and inflammatory factors (IL-13 and IL-17) and help reveal specific pathological factors for diseases associated with airway smooth muscle inflammation on multiomics levels.

## Materials and Methods

### Data

In March 2020, researchers from the University of Chicago released the gene methylation and expression data of ASMCs under the stimulation of multiple inflammatory factors to the Gene Expression Omnibus database (GSE146377) with more than 500 samples (either transcriptomics or methylation data). All the transcriptomics and gene methylation data were generated from the primary cultured ASMCs. In this study, we aimed at interpreting the biological significance of lung smooth muscle and related inflammatory factors during the initiation and progression of multiple diseases like COVID-19 which has ravaged all over the world recently. Following the goal, we downloaded the methylation and gene expression profiles of primary cultured ASMCs exposed to IL-13, IL-17, IL-13 + IL-17, and vehicle from the Gene Expression Omnibus database under the accession number of GSE146377. Only samples with methylation and gene expression data were analyzed. Each of the IL-13, IL-17, IL-13 + IL-17, and vehicle groups had 64 samples. Methylation data were generated with Infinium MethylationEPIC and included 786,326 probes. The expression levels of 18,279 genes were profiled with Illumina HumanHT-12 V4.0 expression beadchip. We aimed to investigate the responsive genes of ASMCs to IL-13, IL-17, and IL-13 + IL-17.

### Monte Carlo Feature Selection

The methylation and gene expression profiles of ASMCs have much more features than samples. The Monte Carlo feature selection (MCFS) (Dramiñski et al., 2007) was deemed to be excellent in tackling such type of dataset. It is a powerful and widely used feature selection technology.

To evaluate the importance of features, MCFS generally includes the following steps: (i) the selection of random feature subsets with *m* features from the original whole *M* features (*m* « *M*); (ii) the learning of a classification model on the bootstrap dataset for each feature subset, which generates *p* decision trees from classification model; (iii) the production of *p* × *t* decision trees by repeating the above steps *t* times; and (iv) the calculation of the relative importance score (RI) for each feature. Among the constructed *p* × *t* decision trees, a given feature may occur in some of them. The split on a node using such feature in each of these decision trees can reflect its importance, which can be measured by the information gain achieved by such split. Furthermore, the classification ability of the decision tree should also be included. Thus, the contribution of a feature in a decision tree can be the determined by the information gain achieved by the split, the number of samples in the split node and the classification ability of the tree. The RI value of a feature *f* can be the sum of contributions on all constructed decision trees, which is defined as

(1) $$R\,I_{f}=\sum_{\tau =1}^{p\,t}{\left(w\,A\,c\,c\right)}^{u}\,\sum_{n_{f}\,\left(\tau \right)}I\,G\,\left(n_{f}\,\left(\tau \right)\right)\,{\left(\frac{\mathrm{no}.\mathrm{in}\,n_{f}\,\left(\tau \right)}{\mathrm{no}.\mathrm{in}\,\tau }\right)}^{v}$$

where wAcc is the weighted accuracy, and *n*_*f*_(τ) is a node of feature *f* in the decision tree *τ*. The information gain of *n*_*f*_(τ) is expressed as *IG*(*n*_*f*_(τ)), and (*no*.*inn*_*f*_(τ)) is the number of training samples in *n*_*f*_(τ). *u* and *v* are two weighting factors, which is suggested to one.

After all investigated features are assigned the RI values, a feature list is produced by the decreasing order of RI values of features. In this study, we adopted the MCFS program downloaded from http://www.ipipan.eu/staff/m.draminski/mcfs.html. For convenience, default parameters were used.

### Incremental Feature Selection

Incremental feature selection (IFS) (Liu and Setiono, 1998) is an iterative feature selection approach, which can find the best number of features for a given classification algorithm. For a feature list (e.g., a list produced by the MCFS method), IFS always generates lots of feature subsets, each of which contains some top features in the list. For example, the first feature subset contains the top one feature in the list, the second feature subset consists of the top two features, and so forth. Then, for each feature subset, a classifier can be built based on a given classification algorithm and samples represented by features in the subset. Finally, all constructed classifiers are evaluated by a cross-validation method (e.g., 10-fold cross-validation) (Kohavi, 1995). The classifier with the best performance is extracted, which were called the optimum classifier in the study. Furthermore, the corresponding feature subset was termed as the optimum feature subset.

### Classification Algorithm

As mentioned in section “Incremental Feature Selection,” a powerful classification algorithm is necessary for the IFS method. This study tried four classification algorithms: random forest (RF) (Breiman, 2001), support vector machine (SVM) (Cortes and Vapnik, 1995), k-nearest neighbor (kNN) (Cover and Hart, 1967), and repeated incremental pruning to produce error reduction (RIPPER) (Cohen, 1995). Their brief descriptions are as follows.

#### Random Forest

Random forest (Breiman, 2001) is an assemble classification model that is based on multiple decision tree classifiers. Each decision tree is constructed using randomly selected samples and features. Although decision tree is a relative weak classification algorithm, RF is much power and always an important choice for building different classification models (Tang et al., 2018; Baranwal et al., 2019; Zhao et al., 2019; Jia et al., 2020; Liang et al., 2020). The predicted sample label of RF is obtained on the basis of the aggregated votes of decision tree classifiers. The subtle difference among decision trees in RF causes the potential overfitting of learned models. Thus, RF usually adopts the final consensus results in accordance with the average of all decision trees’ predictions. This study adopted the tool “RandomForest” in Weka (Frank et al., 2004; Witten and Frank, 2005), which implements the RF algorithm. The major parameter, number of decision trees, was set to 10.

#### Support Vector Machine

Support vector machine (Cortes and Vapnik, 1995) is a statistical learning-based classification algorithm. Similar to RF, SVM is another essential candidate for constructing classification models (Sang et al., 2020; Zhou et al., 2020a,b). It first transforms original data from a low-dimensional space to a high-dimensional space by using a kernel function and then divides the data samples of each label in accordance with the principle of data interval maximization in high-dimensional space. It further predicts the new samples’ label in accordance with the interval to which this new sample belongs to. In this work, the tool “SMO” in Weka software (Frank et al., 2004; Witten and Frank, 2005) was employed to construct the SVM classifier. The training procedures are optimized by the sequential minimal optimization algorithm (Platt, 1998). The kernel was a polynomial function and the parameter *C* was set to 1.0.

#### k-Nearest Neighbor Classification

k-nearest neighbor is another classification model with a voting scheme (Theilhaber et al., 2002; Zhang and Srihari, 2004; Yu et al., 2016; Chen et al., 2017a). Given a query sample and one training dataset, kNN includes several computation steps to determine its class: (1) the calculation of the sample distance between the query sample and training samples; (2) the ranking of training samples on the basis of their distances to the query sample; (3) the selection of *k* training samples with the least distance to the query sample (i.e., kNNs, and *k* usually ranges from 1 to 10); (4) the estimation of the label distribution of such *k* nearest training samples; and (5) the prediction of labels for the query sample by using the class label with the highest distribution frequency. In this work, the tool “IBk” in Weka (Frank et al., 2004; Witten and Frank, 2005) was used to build the kNN classifier. The distance between samples was defined as the Euclidean distance.

#### Rule Learning

In addition to the above black-box classification algorithms, we also applied a rule learning algorithm, RIPPER (Cohen, 1995), to generate classification rules for enhancing model interpretation. This algorithm starts to generate rules for the class containing least samples. When a rule is produced, covered samples are removed. Other rules are yielded on the rest samples. Each rule generated by RIPPER is represented by an IF–ELSE statement. For instance, If (GPR44 ≥ 7.200) and (ZC3H12A ≤ 8.211), THEN class = IL-13. Rules in such form can provide human-readable predictions for new samples. In this study, tool “JRip” in Weka (Frank et al., 2004; Witten and Frank, 2005) was utilized to learn RIPPER rules.

### Performance Evaluation

The Matthew correlation coefficient (MCC) (Matthews, 1975; Chen et al., 2017a,b; Zhao et al., 2018), a widely used evaluation measurement, was applied to evaluate the performance of the classification model through 10-fold cross-validation (Kohavi, 1995). MCC ranges from −1 to +1. The classification model with an MCC of +1 has the best performance. Our analyzed data were organized into four categories. Thus, the multiclass version of the MCC (Gorodkin, 2004) was calculated as follows:

(2) $$\text{MCC}=\frac{c\,o\,v\,\left(X,Y\right)}{\sqrt{c\,o\,v\,\left(X,X\right)\,c\,o\,v\,\left(Y,Y\right)}}$$

where *X* is a 0–1 matrix indicating the predicted class of each sample, *Y* is a 0–1 matrix representing the actual classes of all samples, and *cov*(⋅,⋅) represents the covariance of two matrixes.

In addition, the accuracy on each category and overall accuracy (ACC) were also calculated to fully indicate the performance of each model.

## Results

**FIGURE 1:**
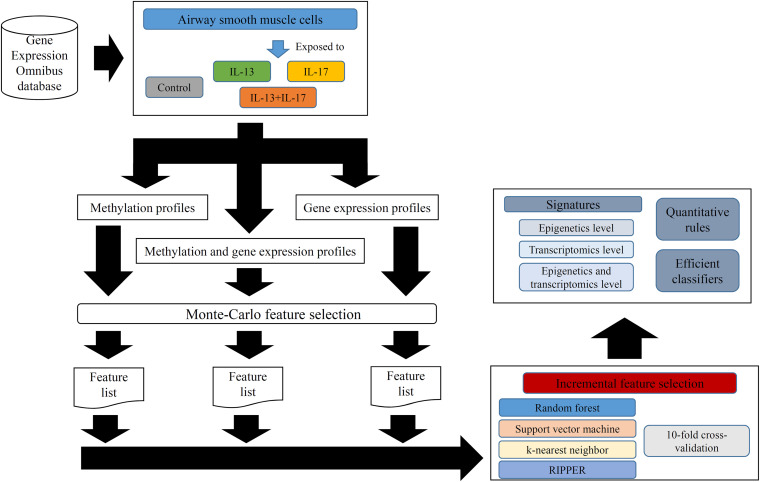
Entire procedures for analyzing methylation and gene expression profiles of airway smooth muscle cells (ASMCs). The methylation and gene expression profiles of ASMCs are retrieved from Gene Expression Omnibus database. The cells are classified into four categories (control, IL-13, IL-17, and IL-13 + IL-17). Three datasets with different combination of profiles are constructed. Each dataset is first analyzed by Monte Carlo feature selection method, producing a feature list. The list is fed into the incremental feature selection method, incorporating one of the four classification algorithms. The results includes: (1) essential signatures from different levels; (2) quantitative rules; (3) efficient classifiers.

In this study, we employed several computational methods to investigate the methylation and gene expression profiles of ASMCs. Samples were divided into four groups: the control group, IL-13 stimulation group, IL-17 stimulation group, and combined (IL-13 and IL-17) stimulation group. We organized the data into three types: the methylation data of the four groups, the expression data of the four groups, and the combined data of the four groups. For each type of data, we utilized a similar analytical pipeline. The entire procedures are illustrated in Figure 1.

### Results for Methylation Data

**FIGURE 2:**
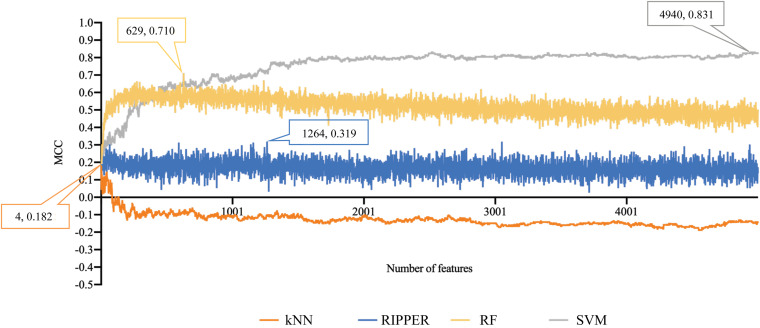
Performance curves of IFS with RF, SVM, kNN, and RIPPER under different numbers of features for methylation data. SVM yields the highest MCC of 0.831.

**FIGURE 3:**
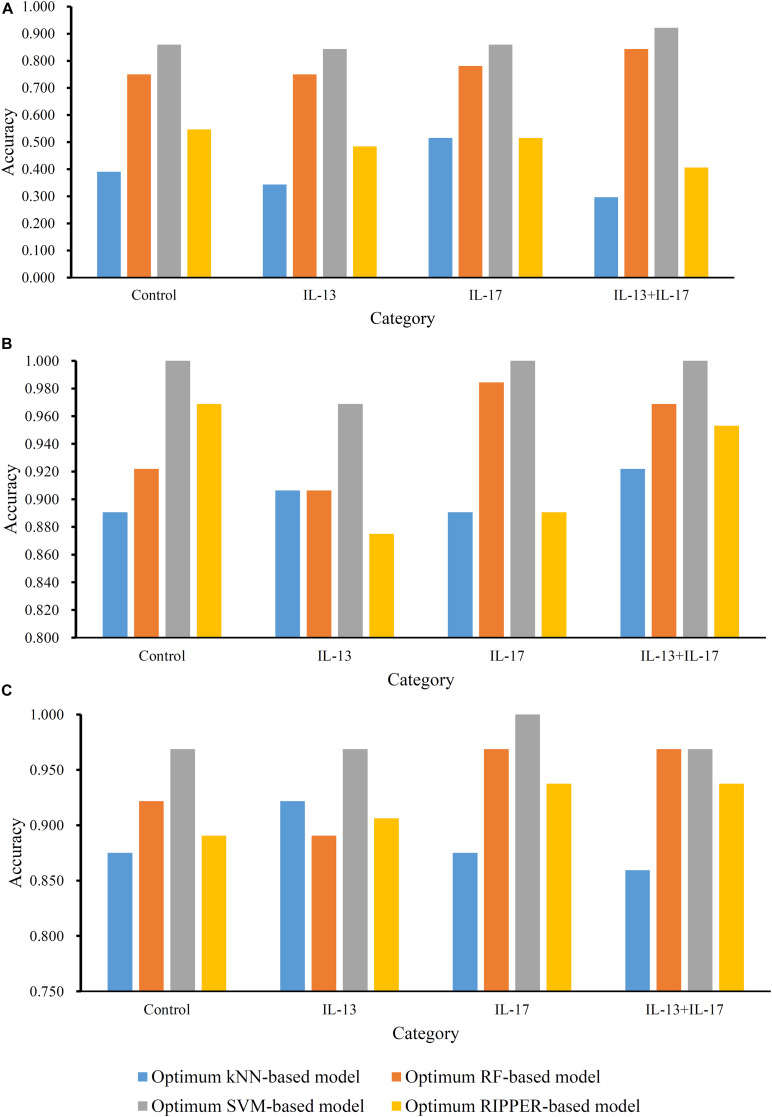
Accuracies on all categories yielded by the optimum classifiers on three datasets. **(A)** Methylation data; **(B)** gene expression data; **(C)** combined data.

For methylation data, we first used MCFS to evaluate each feature, obtaining a feature list, which is available in Supplementary Table 1. Due to the huge number of methylation features, IFS only constructed the top 5000 feature subsets. A RF, SVM, or kNN classifier was built on each feature subset, which was further evaluated by 10-fold cross-validation. The performance of each classifier, including accuracies on four categories, ACC and MCC, is provided in Supplementary Table 2. For an easy observation, a curve with MCC as *Y*-axis and number of used features as *X*-axis was plotted for each classification algorithm, as shown in Figure 2. The SVM exhibited the best performance and had the MCC of 0.831 when top 4940 features were used. For RF and kNN, the best MCC was 0.710 and 0.182, respectively, which was based on top 629 and 4 methylation features. Accordingly, the optimum SVM, RF, and kNN classifiers were built using corresponding optimum feature subsets. The ACCs of these classifiers are listed in Table 1 and the accuracies on four categories are illustrated in Figure 3A. Besides the black-box classifiers, we also tried the rule learning algorithm, RIPPER, in IFS method. Similarly, we still considered the top 5000 feature subsets. The performance of RIPPER classifiers is provided in Supplementary Table 2 and the corresponding curve is shown in Figure 2. The optimum RIPPER classifier yielded the MCC of 0.319 when top 1264 features were used, the corresponding ACC was 0.488 (Table 1). Figure 3A shows the four accuracies on four categories yielded by such classifier. This performance was insufficiently satisfactory for such a rule-based approach.

**TABLE 1:** Performance of the best classification model on three datasets with different classification algorithms.

**Dataset**	**Classification algorithm**	**Number of features**	**ACC**	**MCC**
Methylation	kNN	4	0.387	0.182
	RF	629	0.781	0.710
	SVM	4940	0.871	0.831
	RIPPER	1264	0.488	0.319
Gene expression	kNN	24	0.902	0.870
	RF	40	0.945	0.928
	SVM	3440	0.992	0.990
	RIPPER	794	0.922	0.897
Methylation+ gene expression	kNN	4	0.883	0.844
	RF	96	0.938	0.917
	SVM	3103	0.977	0.969
	RIPPER	42	0.918	0.891

### Results for Gene Expression Data

**FIGURE 4:**
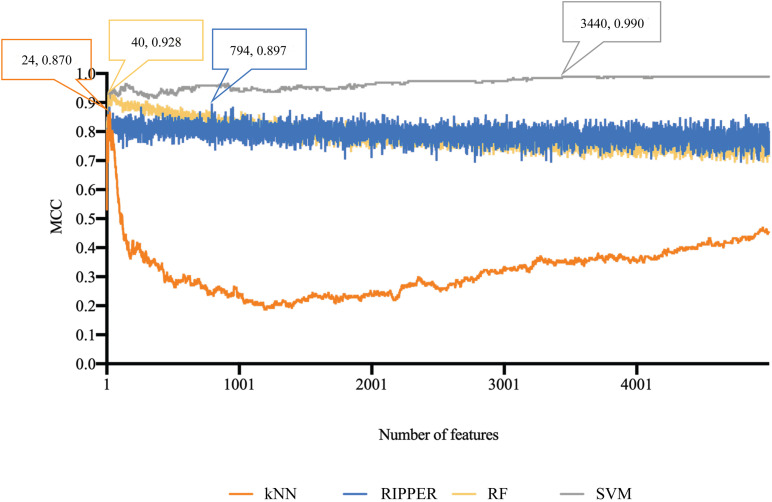
Performance curves of IFS with RF, SVM, kNN, and RIPPER under different numbers of features for expression data. SVM generates the highest MCC of 0.990.

The similar analytical pipeline was applied on the gene expression data. A feature list was first obtained according to the results of MCFS, which are provided in Supplementary Table 3. Then, we applied IFS with 1 as an interval to build classifiers with one of the four classification algorithms. To save time, we still considered top 5000 features. Each classifier was evaluated by 10-fold cross-validation. Obtained measurements are listed in Supplementary Table 4. The corresponding curves were plotted in Figure 4, from which we can see that the four optimum classifiers with different classification algorithms yielded the MCC of 0.870, 0.928, 0.990, and 0.897, respectively, and adopted the top 24, 40, 3440, and 794 features, respectively. The corresponding ACCs are listed in Table 1 and accuracies on four categories are shown in Figure 3B. Similar to the results on the methylation data, the optimum SVM classifier was still best (MCC = 0.990). As for the optimum RIPPER classifier, its performance was much better than that for the methylation data. It produced the MCC of 0.897 and ACC of 0.922 (Table 1). This performance was sufficiently satisfactory. Accordingly, we used top 794 features, which was adopted to build such classifier, to construct rules with RIPPER, obtaining seven rules, where three rules were for IL-13, two rules for control, one rule for both of other two categories. These rules are listed in Table 2. A further analysis would be given in section “Optimal Rules for Distinguishing the Different Statuses of ASMCs.”

**TABLE 2:** Rules by RIPPER on expression data.

**Index**	**Condition**	**Result**
1	(GPR44 ≥ 7.200) and (ZC3H12A ≤ 8.211)	IL-13
2	(SEMA3A ≤ 9.623) and (NFKBIZ ≤ 10.612)	IL-13
3	(MYOM1 ≥ 7.527) and (MAP3K8 ≤ 9.269)	IL-13
4	(NFKBIZ ≤ 10.483) and (MAP3K8 ≤ 8.234)	Control
5	LSS ≤ 10.932	Control
6	CCL26 ≤ 9.291	IL-17
7	Others	IL-13 and IL-17

### Results for Combined Data

**FIGURE 5:**
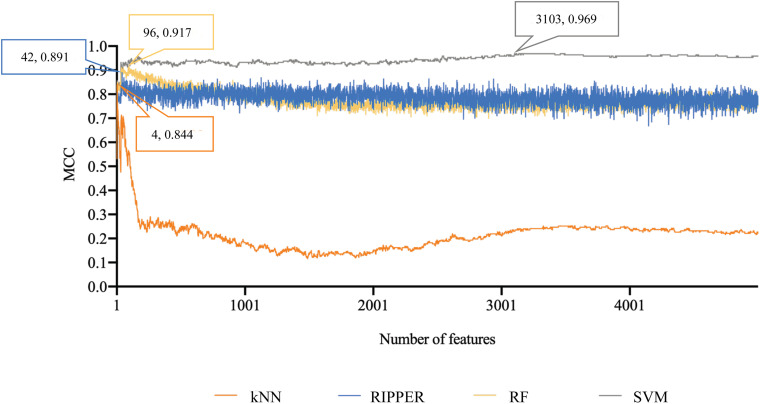
Performance curves of IFS with RF, SVM, kNN, and RIPPER under different numbers of features for combined data. SVM produces the highest MCC of 0.969.

Finally, for combined data, we did the same test. The feature list yielded by the MCFS method is provided in Supplementary Table 5. The IFS method was applied on such list using one of the four classification algorithms. Also, only top 5000 features were considered. The accuracies on four categories, ACCs and MCCs for each classification algorithm are listed in Supplementary Table 6 and a curve for each algorithm was plotted in Figure 5 to show the trends of the performance. It can be observed that SVM consistently achieved the best performance among all algorithms. Its MCC was 0.969 when 3103 top features were used. The ACC was 0.977 (Table 1) and accuracies on all categories are shown in Figure 3C. The performance of other optimum classifiers are listed in Table 1 and Figure 3C. The optimum RIPPER classifier also provided good performance of MCC = 0.891, which used top 42 features. In view of this, we obtained seven rules, listed in Table 3, based on these 42 features and RIPPER. Among these seven rules, two rules were for IL-17, three rules were for IL-13, and one rule was for both of other two categories. We would analyze them in section “Optimal Rules for Distinguishing the Different Statuses of ASMCs.”

**TABLE 3:** Rules by RIPPER on combined data.

**Index**	**Condition**	**Result**
1	(COL17A1 ≤ 7.226) and (NFKBIZ ≥ 10.687)	IL-17
2	(CCDC86 ≤ 9.636) and (NFKBIZ ≥ 10.454)	IL-17
3	(DTNA ≤ 7.176) and (NFKBIZ ≤ 10.496)	IL-13
4	(CCL11 ≥ 12.814) and (MAP3K8 ≤ 9.294) and (SLIT2 ≤ 11.423)	IL-13
5	PPFIBP2 ≥ 9.582	IL-13
6	MAP3K8 ≥ 8.957	IL-13 and IL-17
7	Others	Control

The results for RIPPER indicated that datasets containing only epigenetic data with RIPPER and the MCC of 0.319 might be unacceptable for further analyses and that the use of methylation data might be ineffective for constructing reliable quantitative rule-based models for distinguishing the different statuses of ASMCs. Expression data and combined data could provide an optimal RIPPER MCC of approximately 0.900, validating the reliability and efficacy of the features and rules learned from the two datasets.

### Enrichment Results

For all three datasets, the best optimum classifiers all used SVM as the classification algorithm. In detail, for methylation data, the optimum SVM classifier adopted top 4940 features, while the optimum SVM classifiers used top 3440 and 3103 features, respectively, for other two datasets. Their corresponding genes were called optimum signatures (genes) for the corresponding dataset. To reveal the potential biological functions that optimum genes are correlated with, we performed GO enrichment analyses using R package (*topGO* v2.38.1) on them. The results are provided in Supplementary Table 7. Of the optimum genes on epigenetic and transcriptomics levels, they enriched five and 39 GO terms, respectively, while 68 GO terms were enriched by the optimum genes on both epigenetic and transcriptomics levels. An analysis would be performed in section “Go Enrichment Analyses for Optimal Signatures for Distinguishing the Different Statuses of ASMCs.”

## Discussion

We applied multiple machine learning models to identify potential multi-omics signatures on the epigenetic and transcriptomic levels. By using our newly presented computational methods, we not only identified a group of effective signatures (genes) that were remarkably correlated with the interactions between interleukins (IL-13, IL-17, or their combination) and ASMCs, but also established specific rules to distinguish four ASMC statuses: quiescent, IL-13 activated, IL-17 activated, and IL-13–IL-17 combined activated. Similar signature analyses have been validated under three conditions, i.e., single transcriptomics level, single epigenetic level, and combined transcriptomics and epigenetic levels. All the identified signatures and rules were validated on the basis of recent publications, indicating the efficacy and accuracy of our prediction. Given the limitation of this manuscript’s length, we only chose several typical genes for introduction. The detailed discussion on the signatures and rules is given below.

### Optimal Signatures for Distinguishing the Different Statuses of ASMCs

#### Signatures on the Epigenetics Level

The top-ranked gene in our prediction list obtained from the epigenetic dataset is *BEND6* with specific methylation alterations on the first exon (**cg08811259**). *BEND6* has been widely reported to be functionally correlated with the Notch signaling pathway (Dai et al., 2013). Early in 2008, the Notch signaling pathway was confirmed to regulate the hyper-responsiveness and inflammation of ASMCs (Okamoto et al., 2008) via multiple interleukins, including IL-13 (Lee et al., 2001) and IL-17 (Plé et al., 2015). Therefore, given that the methylation alteration of *BEND6* has been validated to affect the Notch signaling pathway, this methylation probe together with its target gene *BEND6* are potential biomarkers for distinguishing ASMCs with or without interleukin stimulation.

The next probe (**cg26074603**) targets the 5′ UTR of *KCNC2*. This gene is a core regulator of the voltage-gated potassium channel and has been confirmed to participate in the pathogenesis of multiple diseases, including extratemporal epilepsy (Vetri et al., 2020) and spinocerebellar ataxia (Rajakulendran et al., 2013). Moreover, *KCNC2* has been reported to participate in pulmonary neutrophilic inflammation in the lungs and airway; this condition can involve local smooth muscles (Nadadur et al., 2005). Although direct evidence confirming that interleukins may affect the contribution of *KCNC2* to the inflammation of airway smooth muscles does not exist, previous studies have confirmed that *KCNC2* indeed interacts with multiple interleukins, including, IL-13 and IL-1 (Haas et al., 1993), partially validating our prediction.

The next optimal gene on the methylation level is *MAST4*, which is targeted by the probe **cg06040990**. This gene is a microtubule-associated protein kinase (Sun et al., 2006) that has been widely reported to participate in multiple inflammatory-associated biological processes (Gongol et al., 2017; Cortes et al., 2020). *MAST4* is a part of the PTEN signaling pathway (Valiente et al., 2005; Sotelo et al., 2012), which has been confirmed to mediate the IL-13-induced stimulation, hyper-responsiveness, and inflammation, of airway smooth muscles, thus validating this predicted target gene (Jiang et al., 2012). Similar conclusions have also been further validated in later studies (Hu et al., 2014; Khalifeh-Soltani et al., 2018). Therefore, *MAST4* is definitely correlated with the interleukin-mediated stimulation of airway smooth muscles.

#### Signatures on the Transcriptomics Level

Similar to the analyses based on the methylation-level dataset, our other analyses also identified a group of genes (transcripts) that contributes to distinguishing the different statuses of ASMCs. All such genes/transcripts have also been further validated to be effective in accordance with recent publications.

The first gene in our prediction list is *MAP3K8*, a member of the serine/threonine protein kinase family. *MAP3K8* has been confirmed to be associated with typical differential expression levels in systematic neutrophilic inflammation involving airway tissues (Fu et al., 2013). Although no direct reports have confirmed the regulatory roles of IL-13 and IL-17 in *MAP3K8*-medicated airway inflammation responses, *MAP3K8* has been widely reported to perform an interleukin-dependent inflammatory regulatory role during multiple biological or pathological processes (Glossop and Cartmell, 2009; Kim et al., 2014; Sánchez et al., 2017), implying the specific role of such a gene in the different statuses of ASMCs.

The next gene that contributes to cell classification on the transcriptomics level is *CCL26*, a functional secretory factor that contributes to immune regulatory and inflammatory processes in human bodies (Sangaphunchai et al., 2020). This gene has also been reported to be differentially expressed in airway tissues and participates in the inflammatory response in lung and airway tissues during the pathogenesis of asthma (Sangaphunchai et al., 2020). It has been directly reported to be functionally correlated with IL-13 (Higham et al., 2020; Min et al., 2020) and IL-17 (Kamijo et al., 2020; Mamber et al., 2020) in focal regions surrounding airway smooth muscles at the transcriptomics level and is further pathologically correlated with several chronic lung diseases, including chronic obstructive pulmonary diseases (Min et al., 2020). Therefore, given its functional correlation with the potential regulatory effects of IL-13 and IL-17 on airway smooth muscle inflammation, the predicted gene *CCL26* is definitely an effective signature for cell classification on the transcriptomics level.

***CISH***, also known as *SOCS*, is predicted to be important for the classification of ASMCs with different interleukin stimulation statuses. *CISH* is present at specific expression levels in Treg cells in allergic-associated airway inflammation (Zheng et al., 2020), implying the specific regulatory role of *CISH* in airway regional inflammation on the transcriptional level. *SOCS* can also participate in the regulation of human monocyte inflammatory responses involving IL-13 and IL-4 (Wolde et al., 2020), confirming its potential classification capacity at the gene expression level. Summarizing the specific biological regulatory role of *CISH* in airway tissues reveals that *CISH* is a potential regulatory factor of interactions between interleukins and airway smooth muscles on the transcriptomics level.

#### Combinatory Signatures on the Epigenetic and Transcriptomics Level

Epigenetic- and transcriptomics-level data may be applicable for distinguishing different ASMC statuses on the basis of combinatory signatures. Here, we integrated epigenetic- and transcriptomics-level data to identify specific signatures at the dual-omics levels by using our presented computational method. In accordance with the prediction list, most of the top-ranked features are the same as the features identified through the above transcriptomics-only analyses. Therefore, we further discussed the epigenetic contribution of the top three genes that have already been discussed on the transcriptomics level to provide wide and solid literature support.

As discussed above, *MAP3K8* has been validated to be a transcriptomic regulator that can be used to distinguish different stimulation statuses. The abnormal methylation status of this gene is correlated with multiple chronic pathological conditions, such as lung adenocarcinoma (Tsay et al., 2015) and autoimmune lung injuries (Diaconu et al., 2010; Xie et al., 2018). Although no direct evidence has shown that the methylation alteration of *MAP3K8* is functionally correlated with interleukins, such as IL-17, in the inflammation of airway smooth muscles, a recent publication on colorectal cancer has indicated that the methylation of *MAP3K8* controls focal inflammatory responses via the regulation of related interleukins (Hartley, 2020). Therefore, in addition to its unique contribution on the transcriptomics level, *MAP3K8* is an effective epigenetic regulator of interleukin-mediated airway smooth muscle activation.

*CCL26*, the next predicted gene, is ranked second on the transcriptomic level but fourth on the epigenetic level. It is also associated with specific methylation status in lung- and respiratory-related tissues under various pathological conditions, including lung adenocarcinoma (Dong et al., 2020) and asthma (Kim et al., 2020). *CCL26* has been validated to be regulated by specific interleukins, such as IL-13 (Lyles and Rothenberg, 2019), and further studies have validated that the methylation status of *CCL26* is greatly altered during the inflammatory responses of ASMCs under either pathological or physical conditions (Grozdanovic et al., 2019). Therefore, *CCL26* can also be regarded as a methylation signature of interleukin-mediated inflammation involving ASMCs in addition to its role as an effective transcriptomics signature.

Recently, in correspondence with our prediction, a review of the inflammation profiling of asthma involving airway smooth muscles identified *CISH* as a potential methylation biomarker for airway regional inflammation. Furthermore, *CISH* has been reported to exhibit different methylation patterns in different asthma statuses with different interleukin profiles (Vermeulen et al., 2020), validating the specific role of *CISH* in inflammatory lung diseases on the methylation level.

Collectively, all the optimal signatures have been validated even at the dual-omics level by recent publications. Summarizing the classification model of datasets on different levels revealed that the optimal features on transcriptomics level are similar to those based on combinations but different from those on the methylation level, indicating that transcriptomics-level datasets may perform better than other datasets in indicating the different activation statuses of airway smooth muscles under interleukin stimulation.

### Optimal Rules for Distinguishing the Different Statuses of ASMCs

In addition to the above specific signatures for distinguishing the different statuses of ASMCs, we established a group of effective quantitative rules for cell classification by using the RIPPER computational method. In accordance with the above discussion, we focused on the quantitative rules obtained by using transcriptomics-level data and the dataset combining transcriptomics- and epigenetic-level data.

#### Rules on the Transcriptomics Level

We identified seven rules to distinguish the four groups of cells on the transcriptomics level. The first three rules are defined to identify groups under only IL-13 stimulation and involved genes *GPR44*, *ZC3H12A*, *SEMA3A*, *NFKBIZ*, *MYOM1*, and *MAP3K8*. We have already analyzed the specific role of *MAP3K8* in IL-13- or IL-17-stimulated inflammation involving ASMCs (Hartley, 2020). For other quantitative parameters, we took *GPR44* and *MYOM1* as two typical examples. *GPR44* encodes a receptor for prostaglandin D2. *IL13* participates in the activation of Th2 cells, on which our target gene *GPR44* is expressed. Therefore, *GPR44* can be reasonably predicted to have a greater expression level in the group under *IL-13* stimulation (Huang et al., 2016) or at least greater expression than that in the controls and *IL-17* stimulation. Another parameter of MYOM1 is increased expression level in the *IL-13* stimulated group, and we found some evidence confirming that MYOM1 is up-regulated during *IL-13*-mediated interleukin stimulation under inflammatory conditions, (Campbell and Hardman, 2020) although few publications have shown potential correlations between MYOM1- and IL-13-mediated stimulation.

Similar to the rules identified for IL-13 stimulation group, the specific gene *MAP3K8* remains important for low expression levels in control group. A unique parameter, *LSS*, is down-regulated in controls but up-regulated in all activated ASMCs. *LSS* has been widely associated with nonspecific inflammation in human beings (Vykhovanets et al., 2006; Qin et al., 2013; Li et al., 2016). Therefore, interleukin-mediated airway smooth muscle activation can definitely trigger the up-regulation of *LSS*, indicating that the down-regulated expression of *LSS* may be an effective signature for controls without inflammatory reactions on any levels. *CCL26* in the unique rule identifying IL-17 stimulation group is the only quantitative parameter for identifying the *IL-17* stimulated group. As analyzed above, on the transcriptomics level, *CCL26* has been already confirmed to be up-regulated under stimulation by *IL-13* (Wolde et al., 2020). Therefore, the low expression level of *CCL26* may be used to further distinguish samples under only *IL-17* stimulation from samples under combined stimulation. Finally, the remaining samples are reasonably stimulated by *IL-13* and *IL-17*, thus validating the efficacy and accuracy of our quantitative predictive rules.

#### Rules on Epigenetic and Transcriptomics Levels

By combining epigenetic and transcriptomics data, we also obtained a group of combined signatures with specific quantitative thresholds that reflect expression or methylation tendency. In accordance with the combined rules and in correspondence with our above discussion on the comparison of the contributions of methylation and transcription features to cell classification, all the optimal parameters are simply transcriptomics features. The detailed discussion is provided below.

The first two rules identify *IL-17* stimulation group. Both rules include the up-regulation of *NFKBIZ*, a specific regulator of interleukin-mediated immune responses (Garg et al., 2015). Previous studies have already connected the up-regulation of *NFKBIZ* with the stimulation of *IL-17* (Göransson et al., 2009; Chapman et al., 2010). This connection corresponds with our prediction. Another effective parameter, *CCDC86*, is positively correlated with *IFNG* and *IL-13*. Therefore, the low expression level of *CCDC86* may indicate that a group may not be stimulated by *IL-13* and further confirms that a group is stimulated by only *IL-17* but not the combination of interleukins. *NFKBIZ* remains one of the most significant parameters for *IL-17* stimulation group. The up-regulation of *NFKBIZ* indicates the stimulation of *IL-17*. Therefore, the down-regulation of *NFKBIZ* may distinguish this group from the combined stimulation and *IL-17* stimulation groups. In addition, the high expression of *PPFIBP2* is correlated with *IL-13*-associated inflammatory immune responses, and samples not fitting all the above rules can definitely be classified as control group.

### Go Enrichment Analyses for Optimal Signatures for Distinguishing the Different Statuses of ASMCs

As several GO terms were extracted for different datasets, we selected some of them for analysis.

#### GO Enrichment Analyses for Signatures on the Epigenetics Level

As shown in Supplementary Table 7, we only identified five enriched GO terms of different clusters. We chose **GO:0046872 (metal ion binding)** for detailed discussion. For metal ion binding, correlated with ciliary base, calcium ion binding has been shown to regulate the ASMCs related inflammatory reactions via regulating the function of ciliary base (Aisenberg et al., 2016), validating accuracy of the optimum signatures on the epigenetics level.

#### GO Enrichment Analyses for Signatures on the Transcriptomics Level

For specific GO enrichment generated from signatures on transcriptomics level, only 39 enriched GO terms were identified. The detailed results can be seen in Supplementary Table 7. Here, we chose two terms as for detailed discussion: (1) **GO:0005925 (focal adhesion)** and (2) **GO:0001666 (response to hypoxia)**. Early in 2014, a systematic network analyses on the transcriptomics profiling of airway smooth muscle tissue confirmed that focal adhesion associated pathways play irreplaceable role for physical or pathological inflammatory effects like asthma related inflammation (Yick et al., 2014). As for another enriched GO term named as hypoxia, similar with focal adhesion, hypoxia has also been shown to be correlated with the inflammatory activation of ASMCs. Based on related transcriptomics studies (Ricciardi et al., 2008; Yang et al., 2014), hypoxia has been confirmed to be directly correlated with dendritic cell mediated inflammatory responses. Therefore, it is also reasonable for us to enrich our optimum genes at transcriptomics level in such GO term.

#### GO Enrichment Analyses for Signatures on the Epigenetic and Transcriptomics Levels

As shown in Supplementary Table 7, we identified 68 enriched GO terms of different clusters. We chose **GO:0051301 (cell division)** and **GO:0017147 (Wnt-protein binding)** for detailed analyses. For multi-omics data, the GO term seems to be more general. Cell division has been widely shown to be correlated with inflammatory responses in the airway related tissues (McWilliam et al., 1996; Lambrecht et al., 2000; Grausenburger et al., 2010). Therefore, it is reasonable for potential biomarkers distinguishing different ASMCs inflammatory status to enrich in such GO term. As for Wnt-protein binding, WNT and beta-catenin signaling pathway, which involves multiple WNT proteins, has been widely reported to be correlated with the inflammatory responses of ASMC (DiRenzo et al., 2016; Kumawat et al., 2016; Koopmans, 2017).

All in all, as we have discussed above, for the first time, we recognized the functional enrichment pattern of multi-omics biomarkers. Biologically, we identified multi-omics level regulation associated biological entity (functions, processes, or cellular components), laying a foundation for fully demonstration on the inflammatory factor medicated regulations on ASMCs. Methodologically, we confirmed that the application of multi-omics biomarkers for GO enrichment analyses may improve the efficacy and accuracy for disease associated function exploration, providing an alternative approach for pathological studies.

## Conclusion

Via multiple machine learning models, we identified a group of signatures for the different statuses of ASMCs on the transcriptomics, epigenetic, or dual-omics level and established several quantitative rules on the multiomics level for the classification of cells with different biological/pathological statuses. All the qualitative signatures and quantitative rules have been validated by recent publications, confirming the efficacy and accuracy of our analyses. By summarizing the results, we conclude that the use of transcriptomics data may be more appropriate than that of epigenetic data to classify ASMCs under different activation conditions. Moreover, we conclude that the combined use of transcriptomics and epigenetic data is highly effective and accurate for cell classification.

## Data Availability Statement

Publicly available datasets were analyzed in this study. This data can be found here: https://www.ncbi.nlm.nih.gov/geo/query/acc.cgi?acc=GSE146377.

## Author Contributions

TH and Y-DC designed the study. Y-HZ, ZL, and TZ performed the experiments. Y-HZ, LC, and HL analyzed the results. Y-HZ, ZL, TZ, and HL wrote the manuscript. All authors contributed to the research and reviewed the manuscript.

## Conflict of Interest

The authors declare that the research was conducted in the absence of any commercial or financial relationships that could be construed as a potential conflict of interest.

## Supplementary Material

Supplementary Table 1 MCFS-ranked features for methylation data.

Supplementary Table 2 Performance of IFS with RF, SVM, kNN, and RIPPER under different numbers of features for methylation data.

Supplementary Table 3 MCFS-ranked features for expression data.

Supplementary Table 4 Performance of IFS with RF, SVM, kNN, and RIPPER under different numbers of features for expression data.

Supplementary Table 5 MCFS ranked features for combined data.

Supplementary Table 6 Performance of IFS with RF, SVM, kNN, and RIPPER under different numbers of features for combined data.

Supplementary Table 7 GO enrichment analyses results for signatures on the epigenetic level, transcriptomics level, epigenetic and transcriptomics level.
